# Mechanisms of Senescence-Related NKG2D Ligands Release and Immune Escape Induced by Chemotherapy in Neuroblastoma Cells

**DOI:** 10.3389/fcell.2022.829404

**Published:** 2022-03-02

**Authors:** Yan Zhang, Ruimin Hu, Bixin Xi, Dimin Nie, Hanxiao Xu, Aiguo Liu

**Affiliations:** Department of Pediatrics, Tongji Medical College, Tongji Hospital, Huazhong University of Science and Technology, Wuhan, China

**Keywords:** neuroblastoma, senescence-associated secretory phenotype, immune escape, NKG2D, exosome

## Abstract

Chemotherapy-induced senescence promotes immunocyte aggregation in the tumor microenvironment by upregulating the surface expression of activating ligands in cancer cells. However, these senescent tumor cells cannot be completely cleared and can induce tumor recurrence. Previous studiesshowed that soluble natural killer (NK) group 2D (NKG2D) ligands impair the recognition of multiple immune cells. In this study, we established an *in vitro* senescence model using neuroblastoma cells subjected to low-dose Chemotherapeutic drug doxorubicin or the Aurora A inhibitor MLN8237. The results showed that different neuroblastoma cell lines showed increased secretion of the NKG2D ligand MHC class I polypeptide-related sequence A/B (MICA/B) following proteolysis after treatment, with MICA/B subsequently recruited to exosomes to downregulate NKG2D expression in NK cells. Interestingly, disintegrin and metalloproteinase domain-containing 10 (ADAM10) was upregulated in senescent tumor cells, and combined treatment with the ADAM10 inhibitor GI254023X and chemotherapeutic drugs inhibited MICA/B secretion and enhanced recognition and killing by NK cells. Additionally, we found that expression of the long noncoding RNA MALAT1 was significantly increased in senescent neuroblastoma cells, and that MALAT1 served as a sponge for microRNA (miR)-92a-3p to counteract miR-92a-3p-mediated repression of ADAM10 levels. Furthermore, administration of a MALAT1 inhibitor or an miR-92a-3p mimic reduced the MICA/B shedding and enhanced recognition and killing by NK cells. These results confirmed that low-dose chemotherapy induces senescence in neuroblastoma cells, and that senescent tumor cells promote the shedding of the NKG2D ligand MICA/B through the MALAT1/miR-92a/ADAM10 axis, thereby contributing to the formation of a suppressive immune microenvironment and promoting immune escape.

## Introduction

Neuroblastoma is one of the most common extracranial malignant solid tumors during childhood (Gao et al., 2020), and its clinical manifestations are highly heterogeneous among children, with some tumors regressing on their own (Zafar et al., 2021). However, children with high-risk neuroblastoma are often prone to recurrence after chemotherapy (Ackermann et al., 2018), yet the mechanism is not fully understood. Related studies have shown that genotoxic stress by chemotherapy induces, besides apoptosis, senescence (Yang et al., 2020), a cellular stress response, causing tumor cells to enter a prolonged growth quiescent phase and thus inhibit their proliferation (Duy et al., 2021). Senescent cells show phenotypic modifications incorporating developed cell morphology with expanded cytoplasmic granularity and are most generally portrayed by the enlistment of senescence-related beta-galactosidase (SA-β-gal) (Toffalori et al., 2019; Salminen, 2021). Senescent cells continuously secrete a large number of bioactive substances, generally termed senescence-associated secretory phenotype (SASP), which can attract cytotoxic T lymphocytes (CTLs) and natural killer cells (NK) to accumulate to remove senescent tumor cells (Birch and Gil, 2020). However, this inflammatory response does not completely clear the senescent tumor cells, and some of them evade immune surveillance to enter the cell cycle and become an important cause of tumor recurrence (Saleh et al., 2019). Related studies have shown that SASP can maintain the stemness of senescent cells and epithelial-mesenchymal transition to promote tumor progression (Saleh et al., 2020a). The signaling molecules in SASP, such as IL-6 and IL-8, can form a suppressive immune microenvironment and contribute to immune escape (Liao et al., 2020). We therefore propose the hypothesis that chemotherapy-induced senescent neuroblastoma cells can inhibit immune cell recognition and killing through SASP, to survive genotoxic stress and relapse.

Natural killer group 2 member D (NKG2D), which is mainly expressed on NK cells and CD8^+^ T cells (Dhar and Wu, 2018), is an important activating receptor for NK cells that mediates cytotoxic effects on tumor cell clearance by recognizing NKG2D ligands on the surface of tumor cells (Alexandra et al., 2019). MICA, an important NKG2D ligand, is expressed in various tumor cells. miR-20a can affect the sensitivity of NK cells to colorectal cancer by downregulating the expression of MICA (Tang et al., 2019). In addition, A disintegrin and metalloproteinase 10 (ADAM10), a catalytically active member of the ADAM family of proteases, also known as “molecular scissors” (Tosetti et al., 2018), is involved in the cleavage of some NKG2D ligands in various types of cancer cells either in steady-state conditions or in response to a variety of stress stimuli, affecting the normal recognition of NKG2D receptors in tumor cells (Oh et al., 2020; Smith et al., 2020). Thus, NKG2D ligand shedding may be an important strategy of tumor evasion from immune surveillance. Zingoni et al. showed that the chemotherapeutic drugs doxorubicin and melphalan can affect the shedding of MIC molecules in multiple myeloma cells, accompanied by upregulation of integrin and ADAM10 expression. Interestingly, upregulation of ADAM10 is associated with the senescence phenotype (Zingoni et al., 2020).

The mechanism of chemotherapy-induced immune escape in senescent neuroblastoma has not yet been elucidated. In this study, we investigated the shedding of MIC molecules during the senescence of neuroblastoma cells and its effect on immune cells, identified the mechanism of competing endogenous RNA involved in ADAM10 regulation, and proposed the positive effect of combining chemotherapy with metalloproteinase inhibitors or siRNA MALAT1 on increasing immune recognition function. This study suggests a new strategy of suppressing the immune escape phenomenon in senescent cells.

## Materials and Methods

### Cell Culture and Drug Treatment

The human neuroblastoma cell lines IMR-32, SK-N-SH, SH-SY5Y, and SK-N-BE (2) and human embryonic kidney 293T cells were obtained from the American Type Culture Collection (Manassas, VA, United States). IMR-32, SK-N-SH, and 293T cells were cultured in Dulbecco’s modified Eagle medium (DMEM, Gibco, Carlsbad, CA, United States) containing 10% fetal bovine serum (FBS, Gibco) at 37°C with 5% CO_2_. SH-SY5Y and SK-N-BE (2) cells were cultured in minimum essential medium/F12 medium (Gibco) at 37°C and 5% CO_2_ until reaching 70% cell density. The neuroblastoma cells were inoculated at 3 × 10^5^ cells/mL in six-well plates, with 0.5 μM doxorubicin (DOX, MedChemExpress, Monmouth Junction, NJ, United States) and 2 μM MLN8237 (MedChemExpress) added after 24 h. Two control groups [treated with complete medium and dimethyl sulfoxide (DMSO)] were established and incubated for 72 h for the cellular senescence model.

### Enzyme-Linked Immunosorbent Assay

Commercial sandwich ELISA kits (BOSTER, Wuhan, China) were used to quantify MHC class I polypeptide–related sequence A/B (MICA/B) levels. MICA/B standards were diluted to different concentrations according to manufacturer instructions, and supernatant was collected from cells cultures after 72 h and added along with the standards to 96-well ELISA plates for a 1.5-h incubation at 37°C. The MICA(1:100, EK0812-CAP, BOSTER) or MICB(1:100, EK0963-CAP, BOSTER) monoclonal antibody was then added and incubated for another 1 h at 37°C, followed by washing the cells three times with TBS, addition of the diluted peroxidase complex, and incubation at 37°C for 30 min. After washing five times, 3,3′,5,5′-tetramethylbenzidine luminescent substrate was added, and color development was performed for 30 min. The optical density at 450 nm was then detected using an enzyme marker.

### Flow Cytometry

IMR-32 cells were transfected or not transfected with si-MALAT1 or miR-92a-3p inhibitor and treated with MLN8237 for 72 h. Each group of cells was digested with trypsin, separated into single-cell suspensions in flow tubes, and washed twice with phosphate-buffered saline (PBS)+2% FBS to detect MICA/B expression by flow cytometry (cytoFLEX, Beckman). Cell concentrations were adjusted to 1 × 10^6^ cells/mL, and 1 µL of either the MICA/B antibody (1:100, Lot#320907, BioLegend) was added to each tube and incubated for 30 min at 4°C in the dark. Cells were then washed twice with PBS+2% FBS and detected by flow cytometry. Exosomes isolated from untreated IMR-32 cells and MLN8237-treated cells were co-cultured with natural killer (NK) cells for 24 h, after which NK cells were harvested and incubated with anti-CD56 (1:100, Lot #362545, BioLegend) and anti-NK group 2D (NKG2D, 1:100, Lot #320807, BioLegend) for 30 min at 4°C in the dark. After washing with PBS, the cells were analyzed using flow cytometry.

### Cell Cycle Analysis

IMR-32 cells were treated with 2 μM MLN8237, 0.5 μM DOX, DMSO, or no treatment as a control, and each group above was collected in flow tubes following trypsin digestion and washed twice with PBS. After discarding the supernatant, anhydrous ethanol was added for overnight fixation at −20°C. The fixed cells were then washed twice with PBS, and 2 µL RNaseA was added for incubation at 37°C for 30 min and then 15 min in a water bath. A propidium iodide staining solution (BOSTER) was then added to the cells, which were analyzed by flow cytometry.

### Senescence-Associated β-galactosidase Staining Assay

The DOX- or MLN8237-treated IMR-32 cells and control groups were fixed at room temperature for 15 min after discarding the supernatant, and the fixative was washed away with PBS. The staining solution was prepared according to the instructions of the SA-β-gal staining kit (Solarbio, Beijing, China), with 930 µL of reagent A, 10 µL of reagents B and C, and 50 µL of X-Gal staining solution added to each well, mixed thoroughly, and reacted overnight at 37°C. The cells were subsequently observed under a microscope (BX41, Olympus).

### Western Blot

To each group of cells or exosome samples, radioimmunoprecipitation assay lysis buffer containing protease inhibitor was added and incubated on ice for 30 min. The bicinchoninic acid method was used to detect protein concentration (AR0197, BOSTER, Wuhan, China). For western blot, 20 µg of each protein was electrophoresed and then transferred to a polyvinylidene fluoride membrane, which was incubated with Tris-buffered saline containing Tween-20 plus 5% skim milk powder for 1 h. Protein bands were isolated according to the molecular weight marker (26616, Thermo Fisher Scientific, Waltham, MA, United States) and incubated with the following primary antibodies: anti-CD63 (1:1000, 55051S, Cell Signaling Technology, Danvers, MA, United States), tumor susceptibility gene 101 (TSG101, 1:1000, 28405S, Cell Signaling Technology), disintegrin and metalloproteinase domain-containing 10 (ADAM10, 1:1000, GTX104940, GeneTex, Irvine, CA, United States), and glyceraldehyde 3-phosphate dehydrogenase (GAPDH, 1:1000, GTX100118, GeneTex). The following day, goat anti-rabbit IgG (1:10000, BA1039, BOSTER) was added and incubated for 1 h in a shaker at room temperature. Blots were then rinsed, and the enhanced chemiluminescence reagent (Sigma-Aldrich, St. Louis, MO, United States) was added for development.

### RNA Isolation and Quantitative Reverse Transcription Polymerase Chain Reaction

TRIzol reagent (TaKaRa, Dalian, China) was used to extract total RNA from each group of cells, and 1 µg of RNA was reverse transcribed to cDNA using the Hifair III First Strand reverse transcription kit (YEASEN, Shanghai, China) according to manufacturer instructions. qPCR was performed using SYBR Green master mix (YEASEN), with *GAPDH* and U6 used as internal references. Three secondary wells were used for each sample, and primer sequences for the genes are listed in Table 1.

**TABLE 1:** Primer sequences used for qRT-PCR.

Gene	Forward primer sequence	Reverse primer sequence
*GAPDH*	5′-GAA​CGG​GAA​GCT​CAC​TGG-3′	5′-GCC​TGC​TTC​ACC​ACC​TTC​T-3′
MALAT1	5′-GCC​ATT​TTA​GCA​ACG​CAG​AA-3′	5′-GAC​AGC​TAA​GAT​AGC​AGC​ACA​ACT-3′
*ADAM10*	5′-TGG​ATT​GTG​GCT​CAT​TGG​TG-3′	5′-GGA​GGA​GGC​AAC​TTT​GGA​TTA​C-3′
U6	5′-CTC​GCT​TCG​GCA​GCA​CA-3′	5′-AAC​GCT​TCA​CGA​ATT​TGC​GT-3′
miR-92a	5′-CAC​TTG​TCC​CGG​CCT​GTA​AA-3′	5′-CAG​TGC​GTG​TCG​TGG​AGT-3′

### Exosome Separation

FBS was centrifuged at 150,000 *g* for 16 h to clear bovine exosomes, after which 10% exosome-free FBS solution was used to configure the DMEM. Cells were cultured for 24 h using complete medium, the supernatant was collected and discarded after 1.5 h, and the precipitate was resuspended in PBS. Different groups of exosome samples were examined by electron microscopy (EM) and nanoparticle tracking analysis (NTA) to determine exosome morphology and size.

### Immunofluorescence

Cells (3 × 10^5^ cells/well) from different treatment groups were inoculated in six-well plates, with non-adhered cells subsequently removed. Adhered cells were washed twice with PBS, fixed with 1 ml of 4% paraformaldehyde at room temperature for 15 min, washed three times with PBS, incubated with goat serum for 1 h and then placed in a wet box overnight with the primary antibody anti-ADAM10 (1:100, GTX104940, GeneTex). After washing with PBS, 4′,6-diamidino-2-phenylindole (DAPI, Beyotime, Beijing, China) was added dropwise and incubated for 5 min at room temperature in the dark, with excess DAPI washed away and the film sealed with a blocking solution containing an anti-fluorescence quencher (Beyotime) for observation under a fluorescence microscope (BX41, Olympus).

### Cell Transfection

Small-interfering (si)RNA targeting the long noncoding RNA (lncRNA) MALAT1 and si-NControl (5′-GCU​CCG​AUU​UCU​CGA​ACA​A-3′ and 5′-GAG​UUG​UGC​UGC​UAU​CUU​A-3′, respectively) were synthesized by Genomeditech (Shanghai, China). The microRNA (miR)-92a-3p inhibitor was obtained from RIBOBIO (Guangzhou, China). Cells (1.5 × 10^5^ cells/mL) were cultured in six-well plates, and fusion was observed at ∼50% confluence after 24 h. The supernatant was then discarded, and 2 ml of DMEM was added. We then dissolved 2 µL of siRNA/miRNA or si-NControl in 200 µL of Opti-MEM (Gibco), followed by the addition of Hieff Trans (6 µL) *in vitro* siRNA/miRNA transfection reagent (YEASEN) was added and incubated for 10 min. The siRNA mixture was then added dropwise to each well and incubated for 48 h for subsequent experiments.

### NK Cell Isolation and Culture

Human peripheral blood (20 ml) was collected in a heparin anticoagulation tube, slowly injected into Ficoll separation solution (#07801, Stem Cell technologies, Vancouver, BC, Canada), and centrifuged at 800 g for 20 min. The middle layer of single nucleated cells was removed, washed twice with PBS, and the concentration was adjusted to 1 × 10^6^ cells/mL. We then added 10 µL of human CD56-positive magnetic beads (130-050-401, Miltenyi Biotec, Bergisch Gladbach, Germany) to the cells for a 15-min incubation at 4°C in the dark, followed by washing with running buffer (130-091-221, Miltenyi Biotec, Bergisch Gladbach, Germany). The LS column (130-090-544, Miltenyi Biotec, Bergisch Gladbach, Germany) was fixed on a magnetic stand, and the cell suspension was added in two separate rounds for elution with 2 ml of running buffer. The collected cells were adjusted to a density of 3 × 10^6^ cells/mL in serum-free medium. We then added interleukin (IL)-2 (200 U/mL, MedChemExpress) and IL-15 (50 ng/ml, MedChemExpress) for continuous incubation for 14 days. The medium was supplemented with fresh medium and cytokines according to the cell density.

### Cytotoxicity Assay

Target cells were digested into a single-cell suspension with trypsin, and 10 µL of calcein-AM (BioLegend) was added and incubated for 30 min in the dark. The concentrations of the prepared effector cells (NK cells) and target cells (IMR-32) were adjusted to 3 × 10^6^ cells/mL and 1 × 10^6^ cells/mL, respectively, and the cells were mixed at effector–target ratios of 5:1, 10:1, and 20:1. Effector cells and target cells were then added to a 96-well plate, and natural-release wells (100 µL target cells +100 µL medium) and maximum-release wells (100 µL target cells +100 µL lysate) were set up, with three replicate wells established for each sample. The 96-well plates were incubated at 37°C for 4 h, after which centrifugation was performed at 800*g* for 10 min, and the supernatant was aspirated into a new 96-well plate. The fluorescence value of the supernatant was detected by excitation at 485/420 nm and emission at 530/525 nm.

### Dual-Luciferase Reporter Assay

Wild-type (WT) or mutant (MUT) dual-luciferase reporter plasmids for *ADAM10* and MALAT1 and containing miR-92a-3p-binding sites (synthesized by Genomeditech) were constructed separately. The plasmids and miR-92a-3p mimics or miR-NC were transfected into 293T cells using Hieff Trans *in vitro* transfection reagent (YEASEN). Luciferase activity was detected using the Dual Luciferase Reporter Assay kit (YEASEN) after 48 h, Renilla luciferase activity was normalized to Firefly luciferase activity for each transfected well.

### Statistical Analysis

Experiments were performed in triplicate, and data were analyzed using SPSS (v22.0, IBM Corp., Armonk, NY, United States). An unpaired two-tailed Student’s *t* test was used to perform statistical comparisons between two groups, and analysis of variance was used for multiple comparisons. Data were presented as the mean ± SD (x ± s). A *p* < 0.05 and considered statistically significant.

## Results

### Chemotherapy-Induced Cellular Senescence Promotes the Release of the NKG2D Ligand MICA/B

**FIGURE 1:**
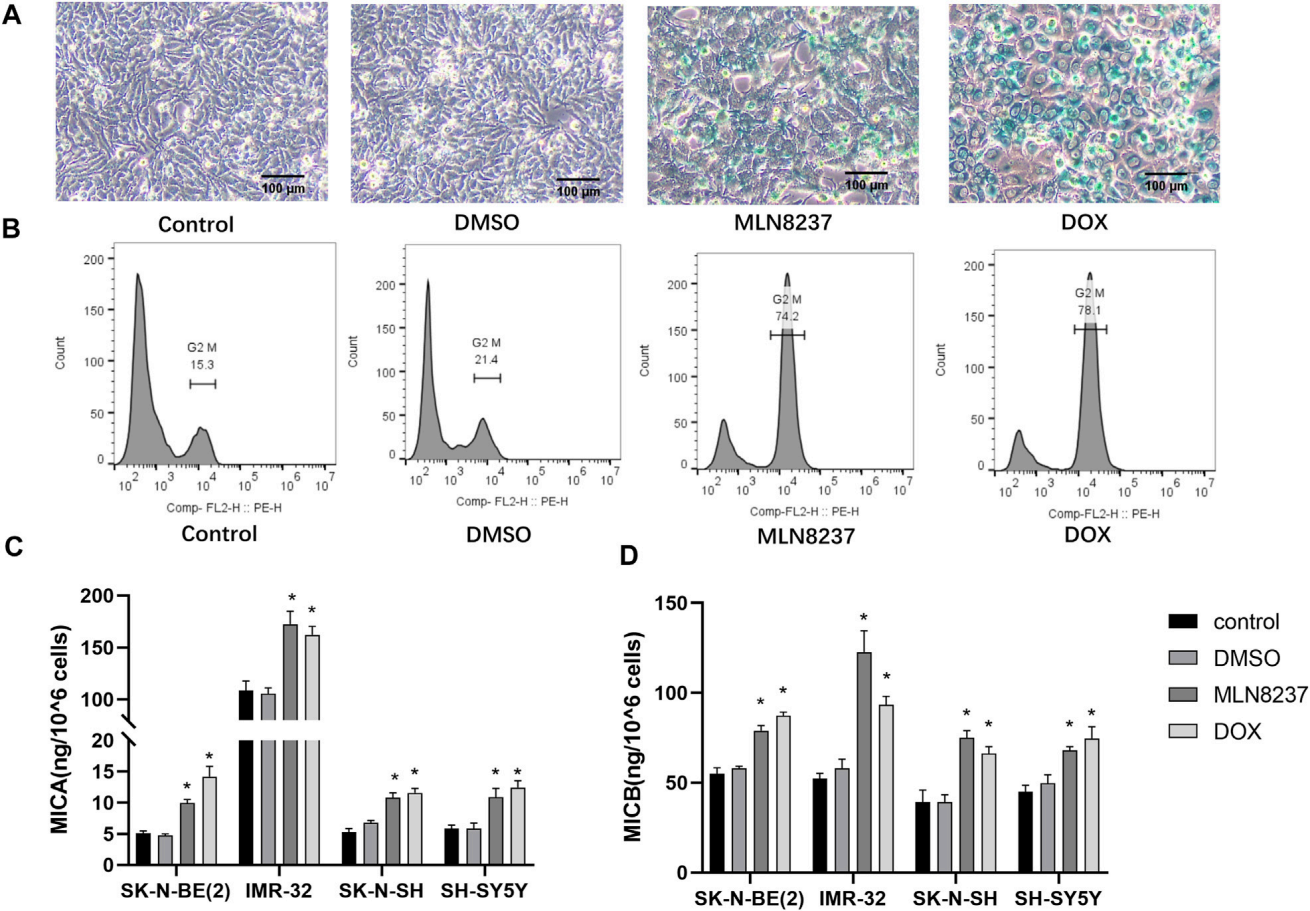
MICA/B release is increased from senescent cells. **(A)** SH-SY5Y cells were treated with 2 μM MLN8237, 0.5 μM DOX, DMSO, or no treatment as a control, and after 72 h, cellular senescence was evaluated by SA-β-gal staining. Senescent cells are shown by blue staining (magnification: ×20). **(B)** Cell cycle progression was assessed by flow cytometry. **(C)** Four different neuroblastoma cell lines were treated with 2 μM MLN8237, 0.5 μM DOX, DMSO, or no treatment as a control, and after 72 h, **(D)** MICA and MICB concentrations in the supernatant were evaluated by ELISA. Data represent the mean ± SEM of three independent experiments. *p < 0.05.

SH-SY5Y human neuroblastoma cells were treated with 2 µM MLN8237 or 0.5 µM doxorubicin for 72 h, the cells were observed to be large and flattened under the microscope. β-galactosidase staining showed that most of the cells in the treated group appeared to be blue-stained (Figure 1A). Meanwhile, the flow cytometry results showed that the cells in the drug-treated group showed cycle arrest and that a significant increase in G2/M phase cells was significantly increased (Figure 1B). The above results indicate that low-dose chemotherapeutic drug stimulation can induce a senescent state in the cells. The release of MICA/B was significantly increased in all four different neuroblastoma cell lines compared to the control group after drug-stimulated senescence (Figure 1C). Because these treatments block cell proliferation, data were normalized taking into account cell num. The concentration of MICA/B in IMR-32 supernatant was much higher than that in other groups, thus, IMR-32 was selected to continue the subsequent experiments.

### Exosome Delivery of MICA/B Inhibits NKG2D Expression in NK Cells

**FIGURE 2:**
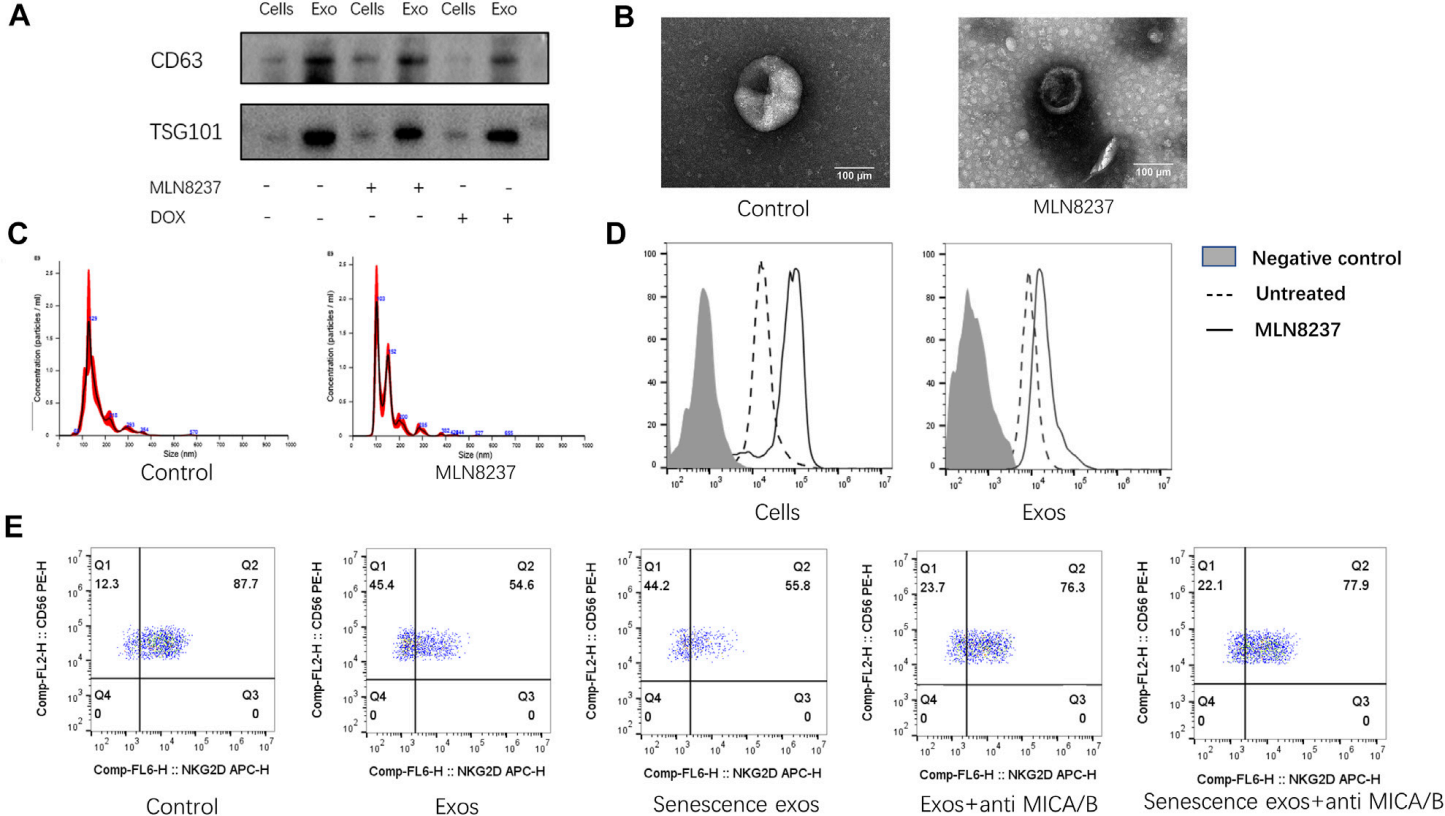
Inhibition of NKG2D expression by exosomal MICA/B. **(A)** IMR-32 cells were treated with 2 μM MLN8237 or 0.5 μM DOX for 72 h. No treatment was used as a control, and exosomes were isolated by ultracentrifugation. Western blot was used to evaluate CD63 and TSG101 levels in whole cell lysates of the cells or isolated exosomes from supernatants. **(B)** Representative EM image of exosomes and showing the typical cup-shaped morphology. Scale bar, 100 nm. **(C)** Analysis of particle size distribution and concentration in exosomes. **(D)** IMR-32 cells were treated with 2 μM MLN8237 for 72 h, no treatment was used as a control. The expression of MICA/B in IMR-32 cells and exosomes were determined by flow cytometry, and exosomes were pretreated by coated latex microbeads. **(E)** Normal human peripheral blood NK cells were used as a control. Exosomes isolated from untreated IMR-32 cells, and MLN8237-treated cells were co-cultured with NK cells for 24 h. Co-cultures were spiked with the MICA/B antibody, and NKG2D expression was detected by flow cytometry.

To confirm the presence of exosomes and estimate the purity of isolation, exosome fractions were evaluated by western blot, NTA, and EM. Western blot results showed the presence of known exosome markers CD63 and TSG101, whereas no significant profile was observed in the cell samples (Figure 2A). We found that normal and senescent exosome samples showed a typical cup-like structure (Figure 2B) with particle sizes ranging from to 50–150 nm under EM (Figure 2C). To detect the expression of senescent cell membranes and exosomal MICA/B, MLN8237 was used to induce senescence in IMR-32 cells, and exosomes were coupled to latex microbeads. IMR-32 exosomes showed expression of MICA/B, matching that of the cell membranes. The expression of MICA/B was increased in senescent cell membranes and exosomes compared with the control group (Figure 2D **)**. We then incubated NK cells with normal or senescent cell exosomes for 24 h to detect the expression of NKG2D, which was used as a control. The results showed that surface NKG2D expression was significantly reduced in exosome-treated NK cells, with no significant difference observed between normal and senescent cell exosomes. To determine whether exosomal MICA/B is involved in the reduced expression of NKG2D on the surface of NK cells, each group of exosomes was pretreated with MICA/B-specific monoclonal antibodies. After blocking exosomal MICA/B, we found that downregulation of NKG2D expression was significantly inhibited (Figure 2E).

### ADAM10 is Upregulated in Senescent Cells

**FIGURE 3:**
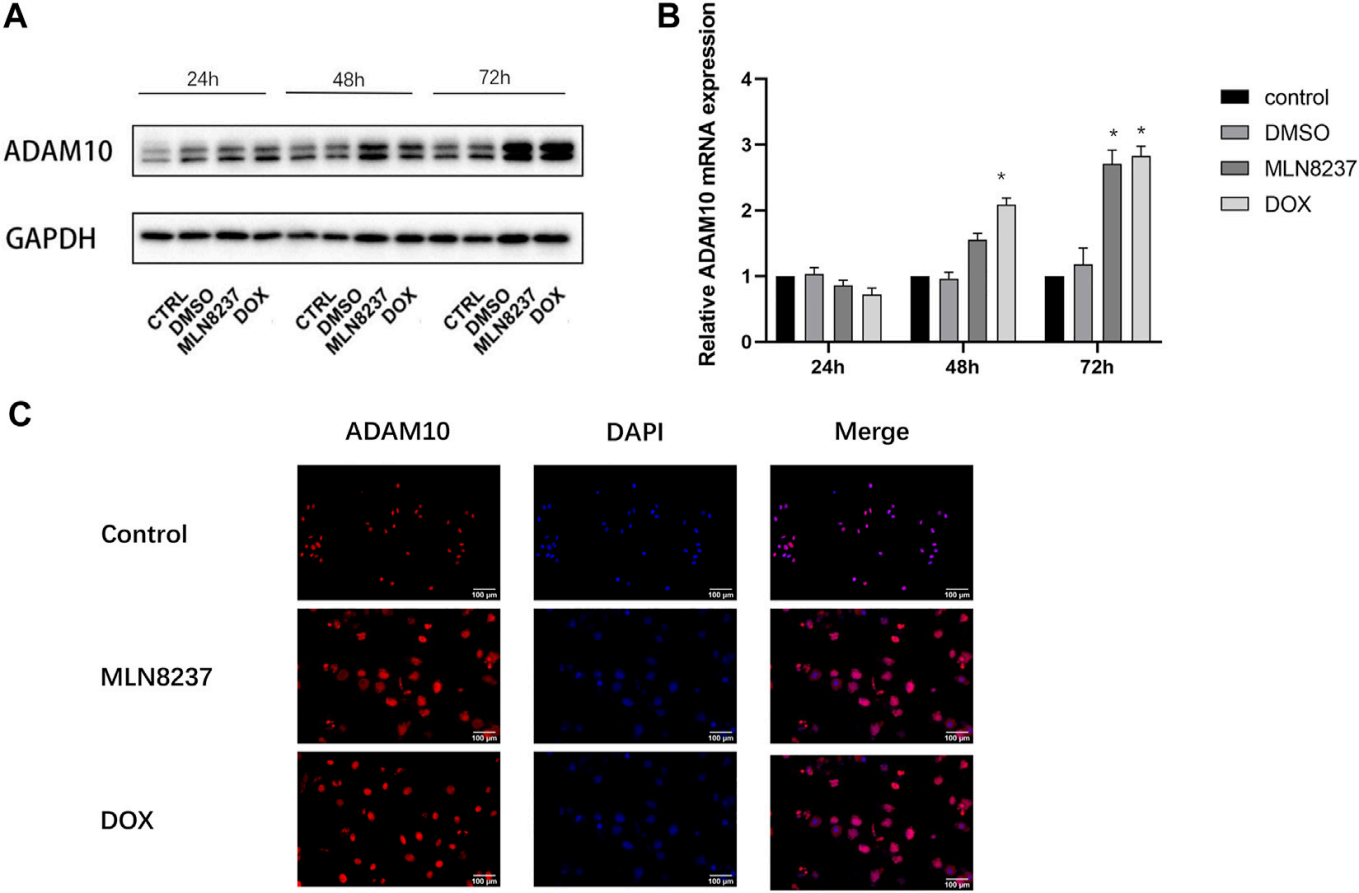
ADAM10 expression is upregulated in senescent cells. **(A)** IMR-32 cells were treated with 2 μM MLN8237, 0.5 μM DOX, DMSO, or no treatment as a control. Cell samples were collected at 24, 48, and 72 h. **(B)** Western blot and qRT-PCR analyses of ADAM10. **(C)** IMR-32 cells were treated with 2 μM MLN8237 or 0.5 μM DOX for 72 h, with no treatment was used as a control. Immunofluorescence detection of ADAM10 expression (magnification: ×20). Data represent the mean ± SEM of three independent experiments. *p < 0.05.

We then investigated the involvement of ADAM10 in the cleavage of MICA/B in senescent cells. Western blot and qPCR results showed that ADAM10 was upregulated at the protein and mRNA levels in IMR-32 cells treated with MLN8237 and DOX for 24, 48, and 72 h, with the most pronounced upregulation observed at 72 h (Figures 3A,B). Immunofluorescence results confirmed ADAM10 upregulation in senescent cells at 72 h along with its distribution in both the nucleus and cytoplasm (Figure 3C). These results suggested that MICA/B secretion by senescent cells might be related to the shearing effect of ADAM10.

### Chemotherapy Combined With ADAM10 Inhibition Reduces MICA/B Release From Senescent Cells to Inhibit Immune Escape

**FIGURE 4:**
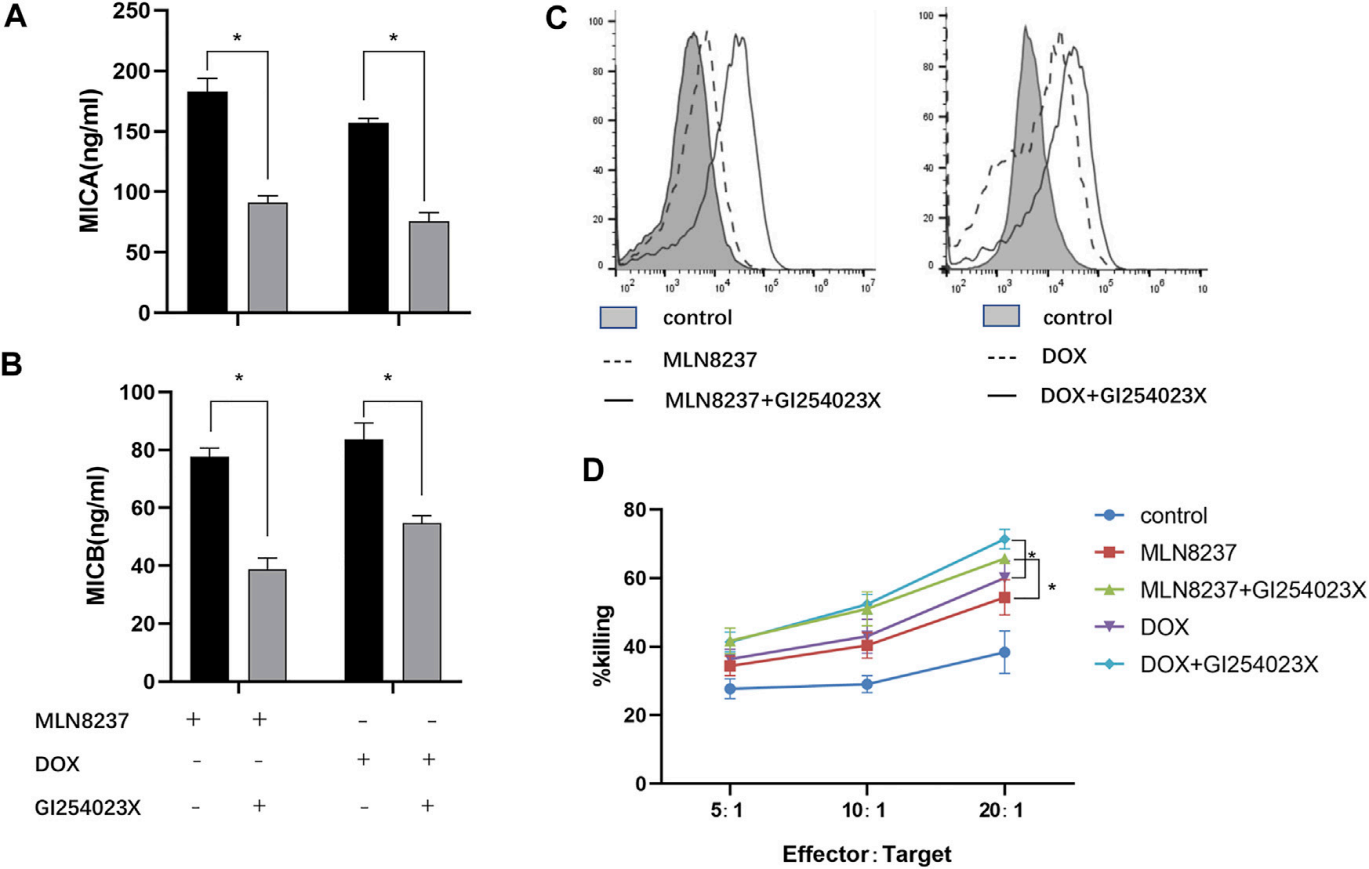
GI254023X combination chemotherapy reduces MICA/B secretion and enhances NK cell recognition. IMR-32 cells were induced to senescence by treatment with MLN8237 or DOX, followed by addition of the ADAM10 inhibitor GI254023X (5 µM) for 72 h. The expression of **(A)** MICA and **(B)** MICB in the supernatant was detected by ELISA. **(C)** Cell-surface expression of MICA/B was detected by flow cytometry following the described treatments. **(D)** Cells in the different treatment groups were used as target cells, and normal human peripheral blood NK cells were used as effector cells. Killing efficiency was detected by the calcein-AM method in co-cultures at effector:target cell ratios of 5:1,10:1, and 20:1 for 4 h. Data represent the mean ± SEM of three independent experiments. *p < 0.05.

We hypothesized that MICA/B shedding from senescent cells occurred via ADAM10-mediated hydrolysis. To test this hypothesis, we confirmed the involvement of ADAM10 in this process through administration of the ADAM10 inhibitor GI254023X (5 μM) to DOX- or MLN8237-treated IMR-32 cells and evaluated MICA/B levels in the supernatants. We observed that this inhibitor resulted in a significant decrease in MICA/B shedding after 72 h (Figures 4A,B), whereas flow cytometry results showed an increase in MICA/B expression on the surface of IMR-32 cells after combination treatment (Figure 4C), thereby confirming the important role of ADAM10 in the release of MICA/B molecules from senescent cells. Notably, compared with cells treated only with the chemotherapeutic drug, cells in the combination group were more easily recognized by NK cells according to presentation of significantly enhanced killing functions, which suggested effective reversal of immune escape in these cells (Figure 4D).

### The lncRNA MALAT1 Regulates ADAM10 Expression Through Competitive Repression of miR-92a-3p

**FIGURE 5:**
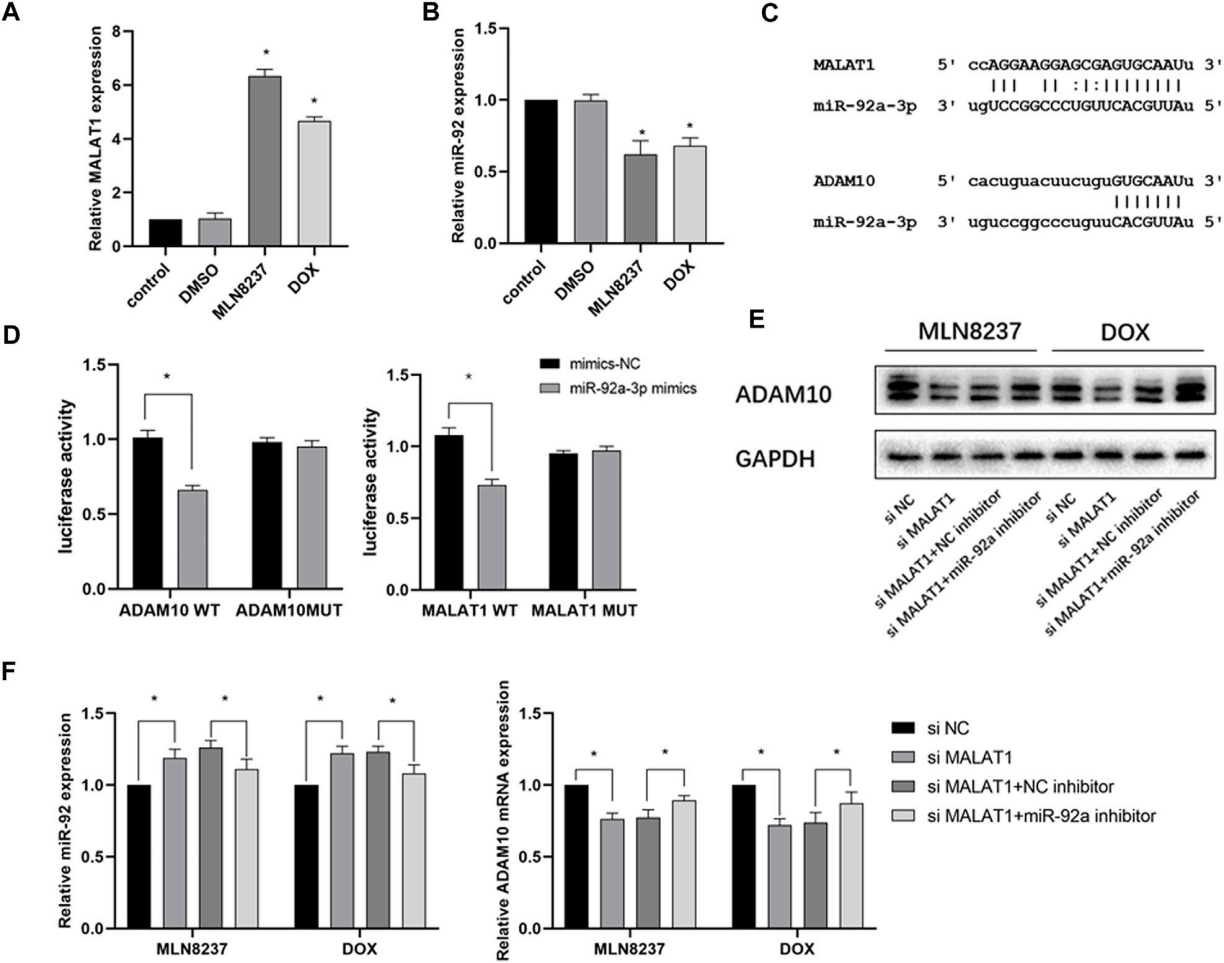
The MALAT1/miR-92a-3p axis regulates ADAM10 expression. **(A)** IMR-32 cells were treated with 2 μM MLN8237, 0.5 μM DOX, DMSO, or no treatment as a control. **(B)** After 72 h, miR-92a-3p and *ADAM10* expression was detected by qRT-PCR. **(C)** Prediction of binding sites between miR-92a-3p and MALAT1 and those between miR-92a-3p and *ADAM10* using ENCORI. **(D)** Confirmation of miR-92a-3p binding to *ADMA10* and MALAT1 was confirmed by luciferase-reporter assay using 293T cells. **(E)** IMR-32 was co-transfected with an miR-92a-3p inhibitor and si-MALAT1, and ADAM10 level was detected by western blot. **(F)** The expression of miR-92a-3p and *ADAM10* was detected by qRT-PCR. Data represent the mean ± SEM of three independent experiments. *p < 0.05.

To explain the changes in ADAM10 expression during cellular senescence, we determined a possible role of noncoding RNA regulation. PCR results showed that MALAT1 expression was significantly upregulated, and that miR-92a-3p was downregulated in IMR-32 cells after 72 h of stimulation with the chemotherapeutic drug (Figures 5A,B). Based on the results predicted from the ENCORI database (https://rna.sysu.edu.cn/encori/tutorialAPI.php), we identified binding sites between MALAT1, miR-92a-3p, and *ADAM10* (Figure 5C). Subsequent dual-luciferase reporter assays indicated that the luciferase activity of the vector containing MALAT1-WT was reduced in cells transfected with the miR-92a-3p mimic, whereas cells containing MALAT1-MUT showed no significant change in luciferase activity. Similarly, in cells transfected with the miR-92a-3p mimic, the luciferase activity of the *ADAM10*-WT-containing vector was reduced, whereas no significant difference was detected in the *ADAM10*-MUT-containing vector (Figure 5D). Additionally, western blot results confirmed that knockdown of MALAT1 in the presence of MLN8237 or DOX significantly reduced ADAM10 protein levels, whereas miR-92a-3p knockdown partially attenuated the effect of MALAT1 knockdown on ADAM10 levels (Figure 5E). Moreover, following transfection of senescent IMR-32 cells (induced by MLN8237 or DOX) with si-MALAT1 with or without an miR-92a-3p inhibitor, qRT-PCR results revealed upregulated miR-92a-3p expression and downregulated *ADAM10* expression in the group transfected with si-MALAT1, whereas these results were partially reversed in the group transfected with si-MALAT1 and the miR-92a-3p inhibitor (Figure 5F). These findings suggested that MALAT1 regulates ADAM10 expression through the competitive inhibition of miR-92a-3p.

### The Dynamic Effect of MALAT1 and miR-92a-3p on MICA/B-Mediated Immune Escape in IMR-32 Cells

**FIGURE 6:**
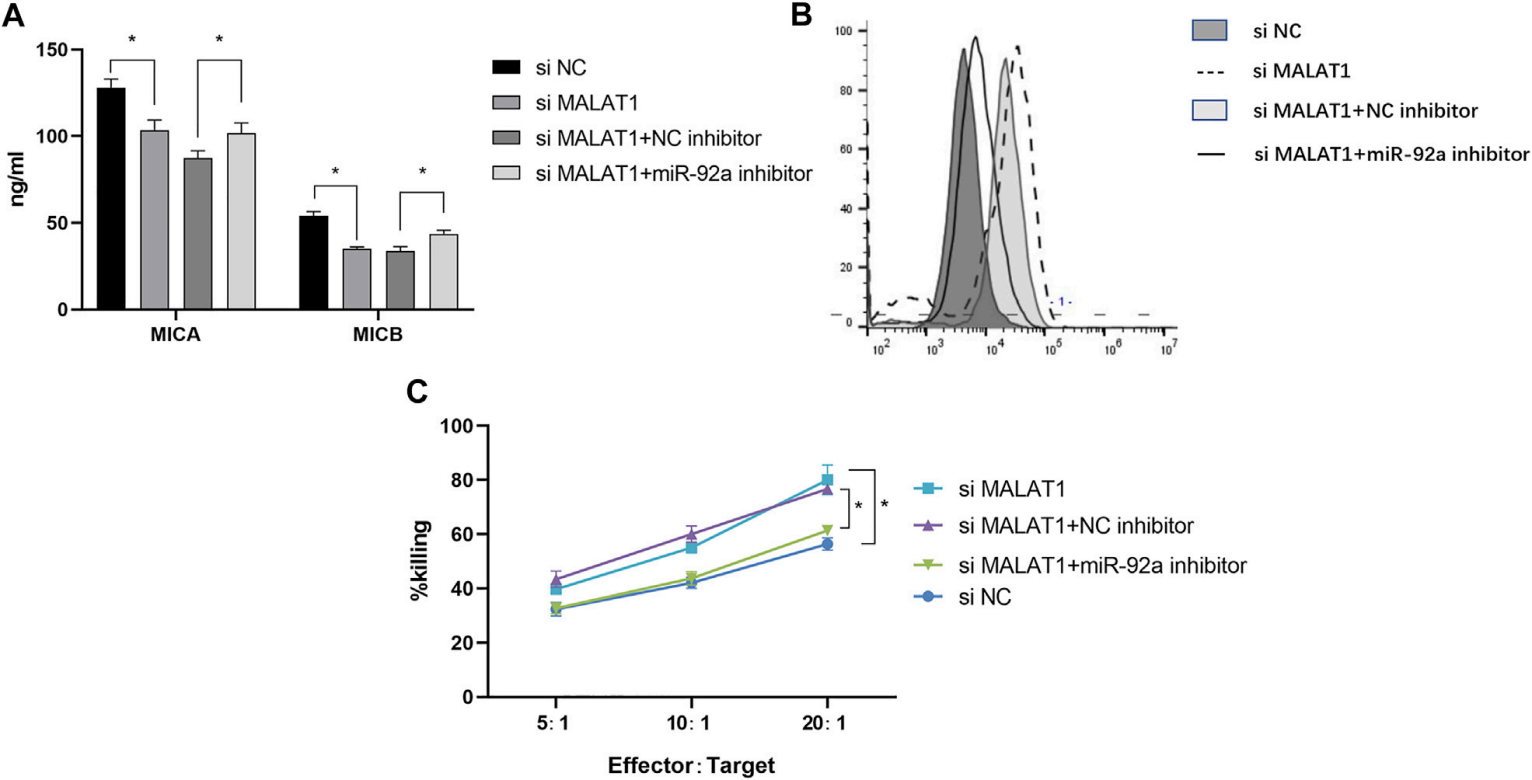
MALAT1 silencing inhibits MICA/B release and inhibits the immune escape of senescent cells. **(A)** Following co-transfection of an miR-92a-3p inhibitor and si-MALAT1 into IMR-32 cells, cells were treated with MLN8237 to induce cell senescence, and ELISA was performed to detect MICA/B levels in the culture supernatant. **(B)** Flow cytometry analysis of MICA/B expression on the cell surface. **(C)** Killing efficiency was detected by the calcein-AM method in co-cultures at effector:target cell ratios of 5:1, 10:1, and 20:1. Cells co-transfected with different vectors were used as target cells, normal human NK cells were used as effector cells. Data represent the mean ± standard error of the mean of three independent experiments. *p < 0.05.

We then evaluated the dynamic effects of MALAT1 and miR-92a-3p on MICA/B-mediated immune escape in IMR-32 cells. Following treatment with 2 μM MLN8237, we found that MICA/B concentration decreased significantly (Figure 6A), whereas MICA/B expression on the surface of IMR-32 cells increased following MALAT1 knockdown or miR-92a-3p inhibition (Figure 6B). Notably, the effect of MALAT1 knockdown was partially attenuated by miR-92a-3p inhibition. Additionally, MALAT1 knockdown enhanced the killing effect of NK cells on IMR-32 cells, whereas miR-92a-3p inhibition exerted the opposite effect (Figure 6C). These data suggested that the MALAT1/miR-92a/ADAM10 axis modulates MICA/B-mediated immune escape in IMR-32 cells.

## Discussion

It has been widely believed that senescence is a stress response that manifests as a state of irreversible growth arrest; however, increasing results have shown that senescent tumor cells have the ability to escape immune surveillance (Prieto and Baker, 2019; Toffalori et al., 2019), survive in the body for a long time, re-enter the cell cycle from a state of prolonged arrest, and maintain a vigorous proliferative potential. Saleh et al. (Saleh et al., 2020b) demonstrated that senescent cells contribute to cancer recurrence and metastasis. In this study, we examined the release of the NKG2D ligand MICA/B in different neuroblastoma cell lines before and after treatment with a low dose of chemotherapeutic drug. The results showed that the level of MICA/B secretion was significantly enhanced in senescent cells, with that in IMR-32 being significantly higher than several other cell lines, which is consistent with previous results in multiple myeloma (Zingoni et al., 2015) and hepatocellular carcinoma (Lazarova and Steinle, 2019). MICA/B is present in NB cell membranes and exosomes, and in addition our study confirmed the relationship between NKG2D ligand release and chemotherapy-induced senescence and suggested that MICA/B may be involved in the formation of an inhibitory tumor microenvironment as an important component of the neuroblastoma SASP. Furthermore, the inhibitory effect of exosomes carrying MICA/B on NKG2D was confirmed by isolating exosomes from the cell culture supernatant, consistent with the results of previous studies (Wensveen et al., 2018; Shyu et al., 2019). There was no significant difference in the roles of exosomes between growing and senescent cells.

Based on the findings of previous studies, it was speculated that the key molecules affecting MICA/B shedding might belong to the metalloproteinase family (Ou et al., 2019). To test this, we performed western blotting, qPCR, and immunofluorescence to confirm that ADAM10 expression gradually increased over time during cellular senescence and was the highest at 72 h. ADAM10, as a hydrolytic enzyme, is involved in the cleavage of various substrates. A previous study showed that ADAM10 expression is related to the senescence phenotype, (Zingoni et al., 2018), and this is consistent with the our results. Based on these results, we combined the metalloprotease inhibitor GI254023X with chemotherapeutic drugs and found that GI254023X inhibited senescence-induced MICA/B release and that more NKG2D ligands were retained on the cell surface, thereby enhancing the ability of NK cells to recognize and kill senescent cells. This finding has potential implications for the clearance of senescent cells induced by chemotherapy and for preventing tumor recurrence during senescence.

Noncoding RNAs play an important role in regulating gene expression, and the prediction of gene binding sites in combination with the lncRNA database revealed that miR-92a interacts with lncRNA MALAT1 and ADAM10. MALAT1 is a widely studied lncRNA in tumors involving pathways such as MAPK/ERK, PI3K/AKT, and β-catenin/Wnt, among others (Wang et al., 2019; Goyal et al., 2021). Its diagnostic and prognostic value in numerous tumors, such as breast, cervical, and colorectal cancers, has been demonstrated (Ma et al., 2019; Xu et al., 2020). However, the relationship between MALAT1 and immune escape remains unknown. In this study, we verified that MALAT1 is physically bound to miR-92a-3p and act as a ceRNA using dual-luciferase reporter assay, and knockdown of MALAT1 in senescent cells was followed by downregulation of ADAM10 expression. At the same time, inhibition of miR-92a reversed downstream ADAM10 expression. This finding further confirms the MALAT1 regulatory network and provides new insight into the immune escape mechanism of senescent cells. Chemotherapy-induced senescence upon MALAT1 knockdown similarly inhibited MICA/B secretion and enhanced NK sensitivity, demonstrating the potential clinical value of clearing senescent tumor cells.

In summary, chemotherapy-induced senescence in neuroblastoma cells inhibits NK cell recognition via direct secretion of MICA/B or delivery of this ligand in the form of exosomes, which acts on adjacent NK cells to form a suppressive immune microenvironment, thus achieving immune escape. This phenomenon is explained mechanistically by the high expression of MALAT1 in senescent cells, which drives ADAM10 expression through the MALAT1/miR-92a/ADAM10 axis and the release of the ligand MICA/B from tumor cell membranes via hydrolysis. Combining low-dose chemotherapeutic drugs with either an ADAM10 inhibitor or silencing of MALAT1 can effectively reverse the shedding of MICA/B, thus enhancing the ability of NK cells to recognize and kill senescent cells and effectively inhibit the immune escape of senescent cells. However, despite these findings, this study was limited to *in vitro* experiments, and *in vivo* experiments have not yet been conducted. In addition, the immune escape mediated by the NKG2D receptor-ligand system does not only exist in NK cells, and the regulatory mechanism of other immune cells expressing this receptor, such as CTLs, still needs further elucidation. The combination chemotherapy regimen proposed in this study has potential clinical implications and may provide a reference for the treatment of neuroblastoma.

## Data Availability

The original contributions presented in the study are included in the article/Supplementary Material, further inquiries can be directed to the corresponding author.

## Abbreviations

ADAM10, disintegrin and metalloproteinase domain-containing 10; DAPI, 4′,6-diamidino-2-phenylindole; DOX, doxorubicin; ELISA, enzyme-linked immunosorbent assay; EM, electron microscopy; FBS, fetal bovine serum; MICA/B, MHC class I polypeptide–related sequence A/B; miR, microRNA; MUT, mutant; NK, natural killer; NKG2D, natural killer group 2D; NTA, nanoparticle tracking analysis; PBS, phosphate-buffered saline; SA-β-gal, senescence-associated β-galactosidase; siRNA, small-interfering RNA; WT, wild type.
